# Low-temperature vacuum permeation of sodium tripolyphosphate and trehalose suppresses the denaturation of myofibrillar proteins in peeled shrimp (*Litopenaeus vannamei*) during frozen storage

**DOI:** 10.3389/fnut.2022.1012864

**Published:** 2022-10-06

**Authors:** Qi Du, Chuangdong Fang, He Qi, Soottawat Benjakul, Santiago P. Aubourg, Bin Zhang

**Affiliations:** ^1^Key Laboratory of Health Risk Factors for Seafood of Zhejiang Province, College of Food Science and Pharmacy, Zhejiang Ocean University, Zhoushan, China; ^2^Pisa Marine Graduate School, Zhejiang Ocean University, Zhoushan, China; ^3^International Center of Excellence in Seafood Science and Innovation, Faculty of Agro-Industry, Prince of Songkla University, Hat Yai, Thailand; ^4^Consejo Superior de Investigaciones Cientificas (CSIC), Inst Invest Marinas, Vigo, Spain

**Keywords:** frozen shrimp, myofibrillar proteins, structure, stability, permeation

## Abstract

Phosphates and trehalose are widely accepted additives in animal muscle products. In this study, the effects of pre-soaking with sodium tripolyphosphate (STPP) and trehalose under vacuum permeation (VP) conditions on the physicochemical properties of shrimp muscle were evaluated over 120 d of frozen storage. The results indicate the STPP/trehalose-VP treatments significantly reduced the thawing loss and prevented changes in the texture, myofibrillar protein (MP) content, and Ca^2+^-ATPase activity of shrimp muscle during frozen storage compared with results of control and individual STPP or trehalose soaking treatments. The histological structure analysis revealed the permeated STPP/trehalose distinctly inhibited the dissociation of muscle fibers and reduced physical damage to connective tissues during storage. Furthermore, analysis of the thermal properties indicated STPP/trehalose treatment increased the $T_{\text{g}}\text{’}$ values of shrimp muscle tissues, likely by restricting the mobility of water molecules in muscle tissues and embedding proteins in the glassy matrix. Thus, the physical destruction caused by ice crystal growth was greatly reduced, due to the absence of water molecules around muscle proteins during frozen storage. Accordingly, the combined STPP/trehalose-VP treatment significantly enhanced the stability of frozen shrimp, and the results support the application of traditional cryoprotective additives. The treated shrimp can be stored at comparatively higher temperatures with limited physicochemical reactions during frozen storage.

## Introduction

Frozen storage is the most common commercial method for the preservation of shrimp and its products. However, freezing and subsequent storage unavoidably and irreversibly ruptures myofibrillar proteins (MPs) and connective structures of shrimp muscle, due to ice crystal growth, solute concentration, protein dehydration, and/or protein and lipid oxidation that occurs in muscle tissues (1, 2). During long-term frozen storage, ice crystals grow gradually at the expense of small ice nuclei and particles, leading to the formation of irregular ice crystals with enlarged size in the extracellular and intercellular space. These crystals cause serious damage to muscle organelles and proteins (3). The physical damage and cold stress greatly affect the function and structure of cytoskeletal proteins (i.e., myosin and its polypeptide chains, actin and its bundling proteins, tropomodulin, tubulin), ribosomal proteins, projectin, calponin, heat shock proteins, and others in tissues. These changes lead to rearrangement, dissociation, aggregation, and irreversible denaturation of muscle proteins during storage (4, 5).

To prevent quality deterioration of frozen muscle products, cryoprotectants, such as phosphates, sorbitol, trehalose, and protein hydrolysate, have been added prior to freezing to maintain the functionality of shrimp muscle during storage. Among these additives, polyphosphates and trehalose have been thoroughly studied and extensively used in frozen shrimp products. Previous studies indicate red shrimp (*P. muelleri*) pre-soaked in 5% (w/v) sodium tripolyphosphate (STPP) solutions showed comparatively low cooking loss and high sensory attributes during frozen storage (6). Chen et al. (7) found that STPP could incorporate into MPs of mantis shrimp (*O. oratoria*) *via* the formation of C-O-P bonds between STPP and serine/threonine residues. This results in increased negative charges and more stable secondary conformation of MPs. Thus, the presence of phosphates in tissues might maintain the functional properties of muscle proteins and slow the degradation of target cytoskeletal proteins (i.e., myosin heavy/light chains, tubulin chains, fast-type skeletal actin), which are advantageous to the stability of shrimp muscle during frozen storage (2). In addition, trehalose can stabilize the biological structures as a protectant against cold stress, oxidation attack, osmotic shock, and other environmental stresses. Importantly, trehalose has a large hydrated volume, which is greatly beneficial in capturing water molecules in muscle tissues and decreasing the content of residual water in the protein matrix. Therefore, trehalose can effectively protect tissues from the destruction caused by ice crystal formation (8). The water-replacement mechanisms were further validated *via* molecular dynamic simulations. The findings indicate trehalose molecules could connect with amino acids *via* hydrogen bonds and substitute part of water around the surface of myosin. The combinations improved the consistency and cooperativity of myosin chains and protected the conformational structure of myosin (9).

Although the applications and mechanisms of phosphates and trehalose have been widely explored in muscle products during frozen storage, advanced methods for cryoprotective preservation of frozen shrimp products are still needed. Few innovative techniques, particularly the improved pre-treatments, have been employed for maintaining the quality and prolonging the shelf-life of shrimp products. The current study was carried out to advance a new approach to maintaining high quality shrimp products. The effects of sodium tripolyphosphate and trehalose soaking under vacuum permeating conditions on the physicochemical properties of shrimp muscle were evaluated with a focus on myofibrillar protein stability during 120 days of frozen storage.

## Materials and methods

### Chemical reagents

Sodium tripolyphosphate (STPP) and trehalose were obtained from Shanghai Aladdin Biochemical Technology Co., Ltd., (Shanghai, China). A bicinchoninic acid assay kit was purchased from Shanghai Saint-Bio Biotechnology Co., Ltd., (Shanghai, China). In addition, an ultratrace Ca^2+^-ATPase activity assay kit was obtained from Wuhan Saipei Biotechnology Co., Ltd., (Wuhan, China). Maleate, tris (hydroxymethyl) aminomethane (Tris), sodium chloride (NaCl), hematoxylin, eosin, Davidson's solution, anhydrous ethanol, and paraffin were provided by Shanghai Macklin Inc., (Shanghai, China).

### Shrimp samples and treatments

Live whiteleg shrimp (*L. vanname*i), measuring 20.5–23.9 g in weight and 12.1–14.2 cm in length, were purchased in July 2021 from a local shrimp farm in Zhoushan, Zhejiang province, China. The collected shrimp were temporarily cultured in oxygenated seawater in a plastic incubator and then transported to our laboratory within 20 min.

Upon arrival, the shrimp samples (800 pieces) were removed from seawater and slaughtered in ice-cold water (0°C) for 10 min. After washing thoroughly with tap water (4°C), the shrimp were manually sorted (according to size), peeled, and beheaded in a cold room (4°C) under sterile conditions. They were not deveined. Next, the peeled shrimp were randomly divided into five groups: control (peeled shrimp soaked in distilled water); STPP group [peeled shrimp soaked in 3% (w/v) STPP]; STPP combined with vacuum permeating [STPP-VP, peeled shrimp soaked in 3% (w/v) STPP maintained at 0.09 MPa in a vacuum chamber] (TZF-6020U, Shanghai Gemtop Scientific Instrument Co., Ltd, Shanghai, China); trehalose group [peeled shrimp soaked in 3% (w/v) trehalose]; and trehalose-VP group [peeled shrimp soaked in 3% (w/v) trehalose maintained at 0.09 MPa in a vacuum chamber]. The concentration of STPP was performed according to our previous results (2). The pre-experiment results (Supplementary Table S1) showed that 0.09 MPa vacuum treatment significantly improved the water-holding capacity of shrimp muscle after 120 d of frozen storage; therefore, the effects of this treatment were further explored in this study. After 3 h of soaking treatments (4°C), the shrimp were removed and drained at 4°C for 3 min. Subsequently, the obtained samples were rapidly placed on plastic trays (25.0 cm × 13.5 cm × 2.3 cm; 50 pieces of shrimp on each tray). The trays were packaged into polyethylene bags (30 cm × 40 cm, 150 μm thickness; not a vacuum packaging; three replicates for each group). Finally, the resulting packaged samples were stored in a refrigerator at −18°C for 120 d. At 30-day intervals, the shrimp samples were thawed at 4°C for 3 h in a refrigerator and subjected to the following analyses.

### STPP and trehalose content determination

Trehalose content in shrimp muscle was measured using an Anthrone colorimetry assay kit (Suzhou Grace Biotechnology Co., Ltd., Suzhou, China) before and after the soaking treatments, according to the manufacturer's instructions. The STPP content in shrimp muscle was determined *via* an ion-exchange chromatography method before and after the soaking treatments, according to the report by Teixeira and Mendes (10).

### Thawing loss determination

Thawed shrimp samples (the weight was measured, defined as *M*_1_; 0.001 g) were placed in a centrifuge tube containing three pieces of filter paper as absorbents. After centrifugation at 4,000 × *g* for 10 min (4°C) (CR7, Hitachi Maxell Ltd., Tokyo, Japan), the samples were collected and weighed again (defined as *M*_2_; 0.001 g). Thawing loss of the shrimp muscle was calculated as follows: thawing loss (%) = (*M*_1_-*M*_2_)/*M*_1_ × 100.

### Texture determination

Textural properties (including springiness and chewiness) of shrimp muscle tissues were measured using a TA.XT PlusC texture analyzer (Stable Micro Systems Ltd., Godalming, UK). The determination was carried out according to the following procedures: pressure holding time, 3 s; cylindrical probe, P/5; measurement location, second abdominal segment; constant speed, 1.0 mm·s^−1^; compression degree, 50% of initial height; and initial trigger force, 0.6 N. Each measurement was executed six times for each group.

### Myofibrillar protein (MP) content and Ca^2+^-ATPase activity determination

MP in shrimp muscle was extracted using Tris–maleate buffer solution, according to the method of Zhang et al. (11). Briefly, mashed shrimp muscle was homogenized (JXFSTPRP-12, Shanghai Jingxin Industrial Development Co., Ltd., Shanghai, China) in the extraction buffer (composed of 0.10 mol/L NaCl and 20 mmol/L Tris-maleate; pH 7.0). After centrifugation at 10,000 × *g* for 15 min [Sorvall LYNX 4000, Thermo Fisher (shanghai) Scientific, Shanghai, China], the obtained precipitate was collected, resuspended in the same buffer, and extracted again. The resulting precipitate was dissolved in Tris–maleate buffer (composed of 20 mmol/L Tris-maleate and 0.60 mol/L NaCl; pH 7.0). MP content in the solution was determined using a bicinchoninic acid assay kit according to the instructions.

MP extractions (1.0–2.0 mg/mL proteins) were combined with 0.6 mol/L KCl solution. Ca^2+^-ATPase activity (μmol Pi/mg/min) in the obtained mixture was determined *via* an ultratrace Ca^2+^-ATPase activity assay kit, according to the manufacturer's instructions.

### Histological structure analysis

Shrimp samples from each group were immersed in Davidson's solution and then transferred to an anhydrous ethanol solution. After washing several times with ethanol, the obtained muscle tissues were embedded in paraffin blocks and sectioned into several slices using a microtome (3000A, Dakewe Biotech Co., Ltd., Shenzhen, China). The obtained sections were stained with hematoxylin-eosin (H&E) solutions and photographed under a light microscope (Xinico Optical Instrument Co., Ltd., Shenzhen, China) (12).

### Differential scanning calorimetry analysis

Glass transition properties of shrimp muscle were investigated using a differential scanning calorimeter (DSC) coupled with a liquid nitrogen cooling system (DSC200F3, NETZSCH Scientific Instruments Trading Ltd., Shanghai, China). The determination was executed according to the method of Sablani et al. (13) with some modifications. Briefly, shrimp muscle tissues (~10 mg) were placed in a perforated aluminum pan using an empty pan as the control reference. The pans were transferred to the DSC cells. The temperature of the samples was maintained at 25°C for 2 min in the presence of high-purity nitrogen (>99.9%) and subsequently cooled from 25 to −80°C and held for 2 min; heated to 30°C and maintained for 2 min; cooled to −35°C (annealing temperature) and annealed for 30 min; cooled to −80°C and maintained for 2 min; heated to 30°C. The cooling/heating rate was 10°C/min during the measurement. The thermodynamic properties (freezing phase transition) of shrimp muscle, including enthalpy change (Δ*H*) and glass transition temperature ($T_{\text{g}}\text{’}$), were calculated from the calorimetry curve recorded by the NETZSCH Proteus^®^ software (NETZSCH Scientific Instruments Trading (Shanghai) Ltd., Shanghai, China).

### Data analysis

Data are expressed as the mean ± standard deviation (SD). All determinations were carried out in triplicate (*n* = 3), except where otherwise specified. *SPSS* software (version 20.0, SPSS Inc., Chicago, USA) was employed to analyze the significant differences calculated from the determinations. Duncan's multiple comparisons were conducted to determine significant differences at the level of *P* < 0.05.

## Results and discussion

### Thawing loss analysis

The changes in thawing loss of shrimp muscle during 120 d of frozen storage are presented in Table 1. Similar increasing trends of thawing loss in the five sample groups were observed during frozen storage, which suggests a notably decreased ability of myofibril to retain water molecules in the three-dimensional structure. Thawing loss significantly (*P* < 0.05) increased from an initial 5.96% (fresh shrimp) to 12.08% after 120 d of storage in the control group, which was much higher (*P* < 0.05) than that of the STPP (7.45%), STPP-VP (6.70%), trehalose (7.52%), and the trehalose-VP (6.72%) treated shrimp samples. These results are partially in agreement with previous reports on the thawing loss of peeled shrimp stored at −18°C for 12 weeks (2).

**Table 1:** Effect of different preservation treatments on the thawing loss (%) of shrimp muscle after 120 d of frozen storage.

**Groups**	**Thawing loss (%) during 120 days of storage**				
	**0 d**	**30 d**	**60 d**	**90 d**	**120 d**
Control	5.96 ± 0.14^bA^	6.77 ± 0.17^bB^	8.89 ± 0.21^bC^	9.80 ± 0.23^cD^	12.08 ± 0.31^cE^
STPP	4.75 ± 0.10^aA^	5.18 ± 0.11^aB^	5.61 ± 0.17^aC^	6.33 ± 0.22^abD^	7.45 ± 0.19^bE^
STPP-VP	4.80 ± 0.12^aA^	5.23 ± 0.14^aB^	5.52 ± 0.19^aC^	6.02 ± 0.15^aD^	6.70 ± 0.11^aE^
Trehalose	4.82 ± 0.13^aA^	5.23 ± 0.13^aB^	5.68 ± 0.20^aC^	6.42 ± 0.14^bD^	7.52 ± 0.20^bE^
Trehalose-VP	4.76 ± 0.11^aA^	5.16 ± 0.12^aB^	5.45 ± 0.18^aB^	6.10 ± 0.13^aC^	6.72 ± 0.14^aD^

The means with different lowercase letters in the same column were significantly different at P < 0.05, while the means with different uppercase letters in the same row were significantly different at P < 0.05.

Moreover, compared with STPP and trehalose groups, the combined STPP-VP and trehalose-VP treatments significantly (*P* < 0.05) decreased the thawing loss of muscle tissues, especially as the storage time progressed (120 d). The STPP and trehalose content in the shrimp samples after the soaking treatments were determined as 0.11 and 0.152 g/100 g muscle, respectively. In contrast, the soaking combined with the vacuum treatment significantly (*P* < 0.05) enhanced the STPP and trehalose content to 0.136 and 0.175 g/100 g in the muscle tissues, respectively. This implied the vacuum treatment was beneficial for the permeation of STPP/trehalose molecules into muscle tissue, resulting in the improved water-holding capacity of shrimp muscle tissues during long-term frozen storage.

STPP and trehalose soaking treatments maintained the water-holding capacity of shrimp muscle tissues during frozen storage, and this was validated in our previous studies (14, 15). Phosphates (e.g., STPP, sodium pyrophosphate, trisodium pyrophosphate, sodium trimetaphosphate) are widely used as generally recognized as safe (GRAS) additives in marine muscle products during pre-treatment processing, and they have demonstrated several beneficial functions in muscle tissues, such as enhancing ion strength, binding with ions in protein complexes, and improving the stabilization of myofibrillar proteins (MPs) (5, 16). Differently, trehalose has a low molecular weight, a large volume of hydration, and high conformational rigidity properties (17). The presence of trehalose molecules in muscle tissues can connect with MPs through electrostatic interactions and form hydrogen bonds. Significantly, they substitute part of water molecules around the surface of MPs (water-replacement hypothesis), stabilizing the fluctuation, flexibility, and structure of protein/polypeptide chains during frozen storage (15). These physicochemical functions help to maintain the water-holding capacity (decreased thawing loss) of muscle tissues, as well as stabilize and protect muscle proteins against physical damage and protein denaturation/oxidation due to ice crystal growth/recrystallization, radical attack, and environmental stress during cold storage (2). In the current study, vacuum-assisted soaking treatments promoted more trehalose/STPP molecules to permeate into the myofilament lattice and intercellular space in the tissues, which were closely connected with the improved water-holding capacity of shrimp muscle, compared with the individual soaking treatments.

### Springiness and chewiness analysis

**Figure 1:**
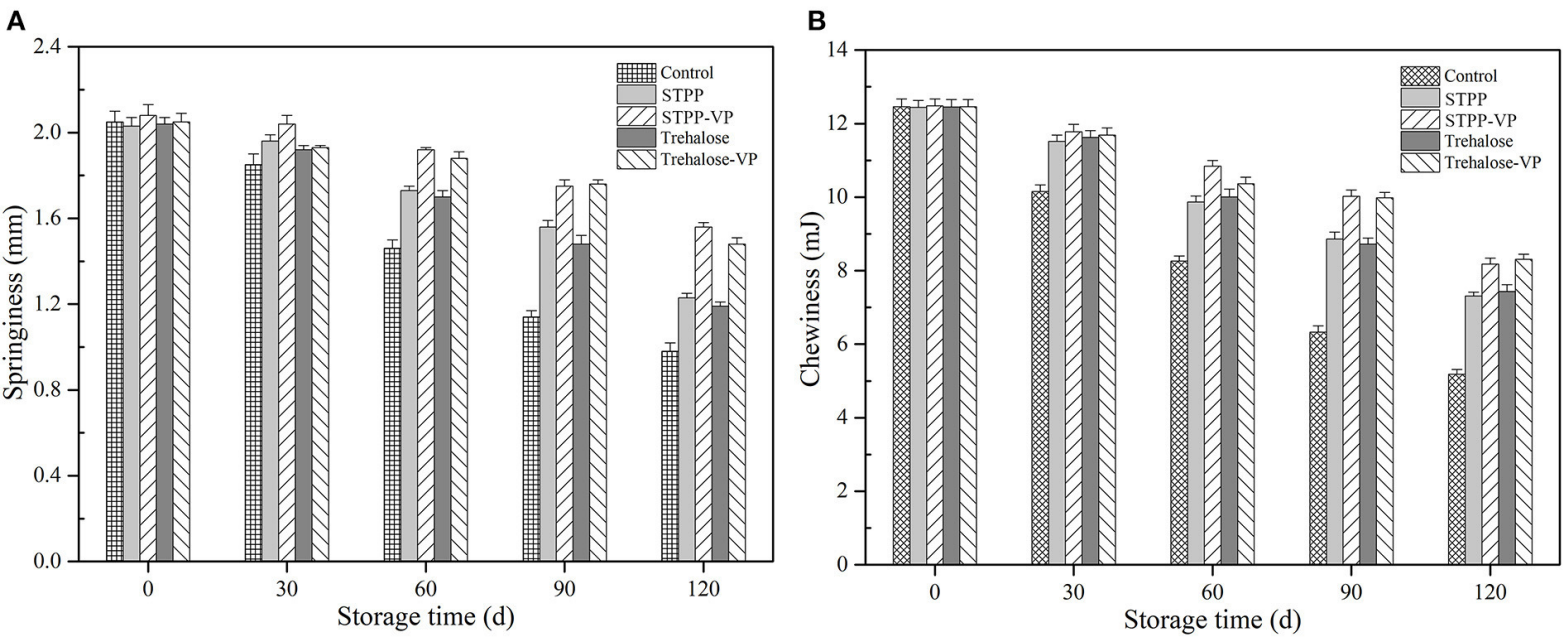
Springiness **(A)** and chewiness **(B)** of shrimp muscle over 120 d of frozen storage for the control, sodium tripolyphosphate (STPP), trehalose, and combined with vacuum permeation (STPP-VP and trehalose-VP) treatment groups.

Textural properties, including springiness and chewiness, of shrimp muscle tissues (Figure 1) were determined after 120 d of frozen storage. Although similar decreasing trends in the textural properties were observed in all shrimp muscle samples, the slope increased rapidly with a prolonged storage period for the control samples. Significant adverse effects on the textural properties were clearly observed in the muscle tissues and were mainly attributed to temperature stress and storage. Compared with the control, the shrimp muscle pre-soaked with STPP and trehalose, respectively, had comparatively higher springiness (Figure 1A) and chewiness (Figure 1B) values. Moreover, the combination treatments of STPP/trehalose and vacuum permeation maintained the springiness and chewiness of shrimp muscle during storage, and the textural properties were significantly better than that of samples treated individually with STPP and trehalose (*P* < 0.05).

The structure, function, and stability of connective tissues and myofibrillar proteins (MPs) are closely linked to the changes in textural properties of shrimp muscle tissue during processing and/or storage. Furthermore, the deterioration of the physical properties was attributed to several factors. Ice crystals grow continuously in muscle tissues. Their shape becomes irregular, and their size increases remarkably during long-term frozen storage, thus leading to considerable mechanical damage to the connective tissues, fiber bundles, and MPs (3). In addition, cathepsins (i.e., calpains and serine proteinases), bacterial activity, and protein/lipid oxidation could also induce the dissociation and fragmentation of MPs and connective tissues (18, 19). In the current study, the soaking treatments combined with vacuum permeation facilitated the transport of more STPP/trehalose molecules into the muscle tissue, and these molecules combined with MPs *via* electrostatic interactions and hydrogen bonds, thereby stabilizing the structure and function of MPs and reducing the physical damage to MPs and connective tissues in absence of partial water molecules around the protein surface (9). Similarly, chitosan combined with STPP nanoparticles was prepared and applied to the shrimp (*L. setiferus*) through a vacuum tumbling treatment (20). The vacuum tumbling treatment significantly maintained the physicochemical properties and reduced the lipid oxidation of shrimp muscle over 120 d of frozen storage. This was primarily due to the improved penetration of saccharide particles into the muscle tissues.

### Myofibrillar protein (MP) content and Ca^2+^-ATPase activity analysis

**Figure 2:**
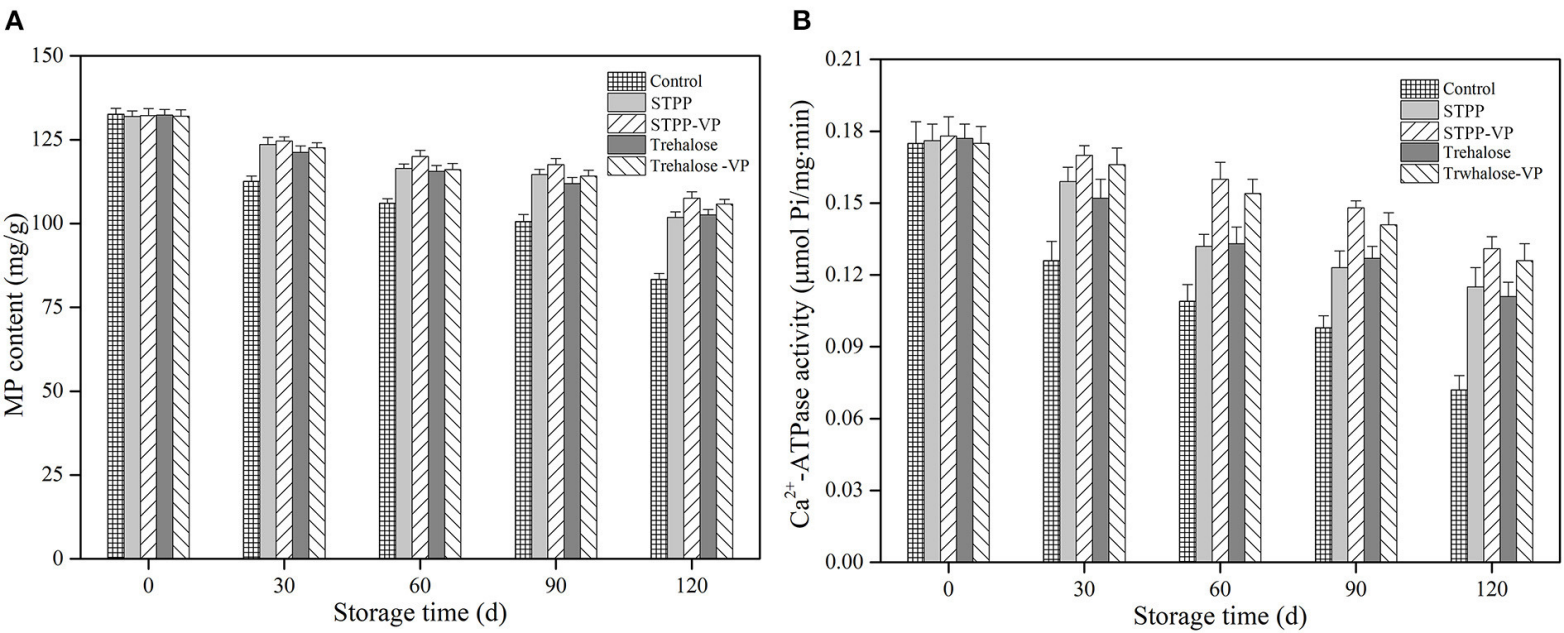
Myofibrillar protein (MP) content **(A)** and Ca^2+^-ATPase activity **(B)** of shrimp muscle treated with the control, sodium tripolyphosphate (STPP), trehalose, and combined with vacuum permeation (STPP-VP and trehalose-VP) during 120 d of frozen storage.

MP content and its Ca^2+^-ATPase activity were measured in the shrimp muscle after 120 d of frozen storage, and the results are depicted in Figure 2. The initial MP content and Ca^2+^-ATPase activity in fresh samples (0 d) were determined as 131.9–132.6 mg/g and 0.175–0.178 μmol Pi/mg/min, respectively. These values indicate good muscle (protein) quality of the shrimp samples. After 120 d of storage, these values significantly (*P* < 0.05) decreased to 83.4 mg/g and 0.072 μmol Pi/mg/min, respectively, in the control samples. Therefore, protein denaturation occurred to some extent in the shrimp muscle tissues, likely resulting from physical damage, protein/lipid oxidation, endogenous enzymes, and their combinations (21). In the case of STPP/trehalose and its combination treatments, the MP content and Ca^2+^-ATPase activity were significantly maintained in shrimp muscle during the entire storage period. After 120 d of storage, the STPP-VP and trehalose-VP treated shrimp showed much higher MP content (107.6 and 105.8 mg/g) and Ca^2+^-ATPase activity (0.131 and 0.126 μmol Pi/mg/min) than those of the control or the individual soaked samples, respectively. These findings further verified the low-temperature vacuum permeating treatments improved the function and stability of MPs in shrimp muscle during long periods of frozen storage.

Protein denaturation is involved in a series of complex physicochemical alternations, i.e., amino acid modifications, peptide dissociation, conformational changes, protein dehydration, and/or radical-induced oxidation, thus resulting in reduced MP content and Ca^2+^-ATPase activity in muscle tissues (22). The normal conformation and structure of MPs are dependent on electrostatic interactions, hydrophobic/hydrophilic interactions, hydrogen bonds, disulfide linkages, van der Waals forces, and their combinations (23). In the current study, the permeated STPP/trehalose molecules interacted with the MPs (i.e., myosin, paramyosin, actomyosin, actin) mainly through the formation of hydrogen bonds, thereby stabilizing the natural structure of MPs in the muscle tissues (9). Furthermore, these small, highly hydrophilic molecules restricted the mobility of water molecules around the surface of MPs, inhibiting the growth and recrystallization of ice crystals and preventing physical damage to muscle fibers and connective tissues (15). These results are in accordance with the water-holding capacity and textural property analyses.

### Histological structure analysis

**Figure 3:**
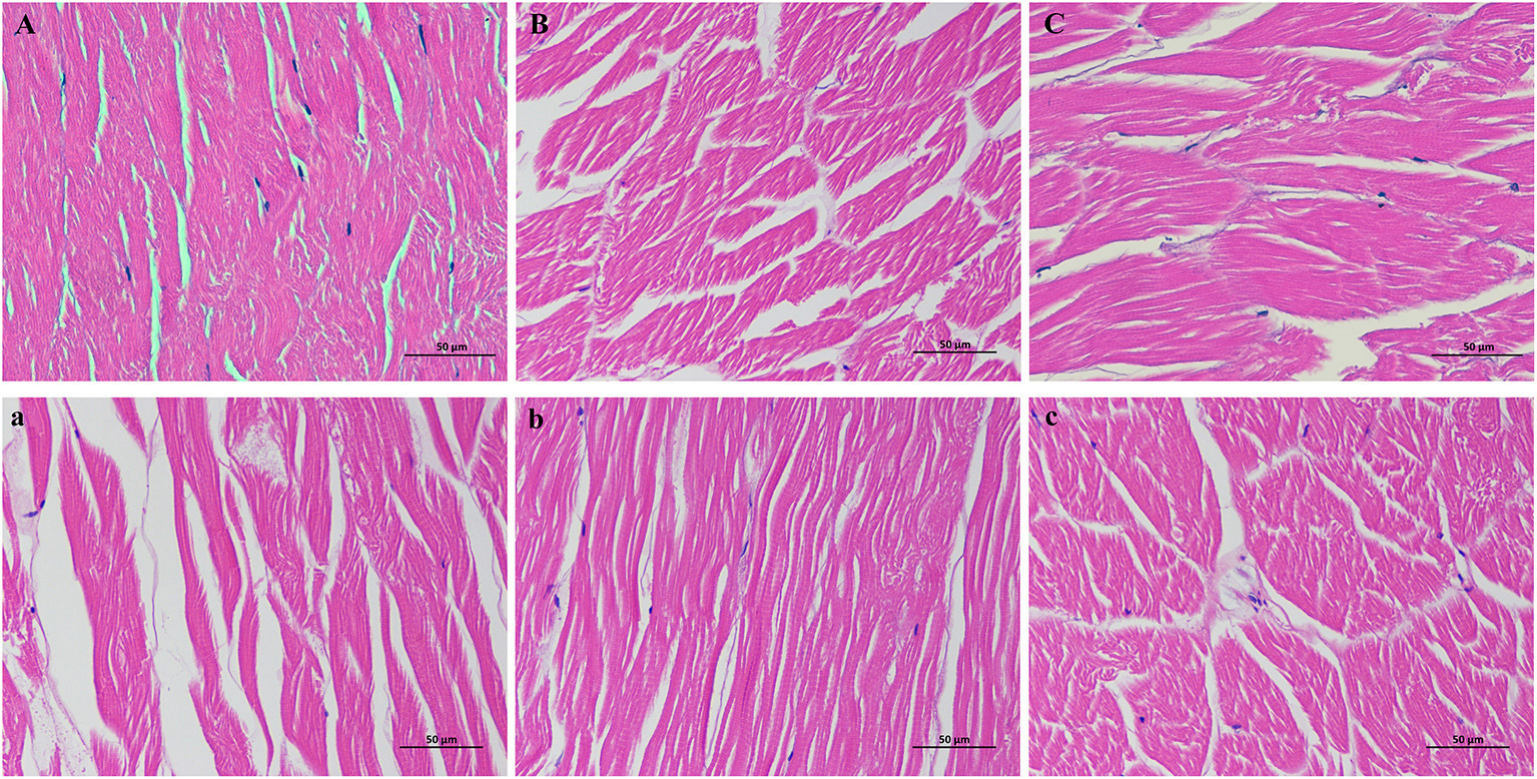
Histological microstructure of shrimp muscle tissues stained with hematoxylin and eosin (H&E) solutions. Control group stored after 0 d **(A)** and 120 d **(a)**, respectively, STPP **(B)** and STPP-VP **(b)** treated shrimp stored for 120 d, and trehalose **(C)** and trehalose-VP **(c)** treated shrimp stored for 120 d.

H&E staining analyses (Figure 3) were performed to visualize the changes in the histological microstructure of the control, STPP, trehalose, STPP-VP, and trehalose-VP treatment groups after frozen storage. For fresh shrimp (Figure 3A), the muscle fibers were tightly connected. In addition, little space and small gaps were observed between fiber bundles. The tissue was well-organized and showed a compact and intact structure. However, after 120 d of storage, the shrimp tissue structure (Figure 3c) was greatly destroyed, most muscle fibers were disrupted, and the space and gaps were distinctly enlarged. This was mainly due to the growth and recrystallization of ice crystals during frozen storage. By contrast, the STPP (Figure 3B) and trehalose (Figure 3C) treatments stabilized the structure of shrimp muscle tissues. The space between fibers and the decomposition of fiber bundles were remarkably smaller than those of the control samples. Furthermore, the histological changes in the STPP-VP (Figure 3b) and trehalose-VP (Figure 3c) treated samples were considerably less than the individual STPP/trehalose soaked samples after frozen storage. This indicates the protective effects of the combined treatment on the physical structure of muscle tissues. These observations are in agreement with the texture and MP content findings.

In the present study, the penetrated trehalose molecules likely connected with MPs and simultaneously substituted water molecules around proteins in the muscle tissues. The reduced number of water molecules slowed the formation, growth, and/or recrystallization of large ice crystals, therefore lessening the extent of disruption and fragmentation of muscle proteins and connective tissues during frozen storage (24, 25). Additionally, the incorporated trehalose molecules could be close to and further embedded into the generated ice crystal interface, partially destroying the natural structure of the crystals and inhibiting the normal growth of ice crystals in the muscle tissues during storage (15, 26).

The alkaline STPP molecules have various functions in muscle tissues, including oxidation inhibition, water-holding, color stability, and protein dispersion properties. Importantly, the penetrated STPP increased the pH of muscle proteins by shifting their isoelectric points, resulting in the swelling of muscle fibers to entrap more water molecules in the muscle proteins (2). In addition, STPP molecules promoted the actomyosin dissociation into actin and myosin through competitive binding with ions in the actomyosin, thus leading to the depolymerization of thin and thick filaments and an increase in water molecule immobilization in the tissues. The chelating properties of STPP also showed some antioxidant effects on muscle proteins and lipids (16). All these properties are beneficial for preventing muscle protein dehydration during frozen storage and preventing the growth of extracellular ice crystals. The results are consistent with our previous studies on frozen shrimp muscle pre-soaked with phosphates (2, 3, 5).

### Glass transition temperature analysis

The thermal properties (Δ*H* and $T_{\text{g}}\text{’}$) of shrimp muscle with different treatments were analyzed, and the results are shown in Table 2. Compared with the control samples ($T_{\text{g}}\text{’}$ = −69.1°C), the shrimp muscle soaked with STPP/trehalose ($T_{\text{g}}\text{’}$ = −57.6°C/−58.6°C) showed higher $T_{\text{g}}\text{’}$ values, suggesting STPP/trehalose molecules had greater cryostabilizing effects on frozen shrimp. Moreover, the combination treatment of soaking and vacuum permeation ($T_{\text{g}}\text{’}$ = −53.4°C/−54.4°C) further enhanced the $T_{\text{g}}\text{’}$ values of shrimp muscle, compared with the individual soaking treatments (*P* < 0.05). The Δ*H* values of shrimp samples showed the opposite trends, representing the heat energy variation in muscle tissues during the freezing phase transition. The Δ*H* values of shrimp treated with STPP-VP and trehalose-VP were determined as 168.9 and 170.3 J/g°C, respectively, which were significantly (*P* < 0.05) lower than those of the single treatments (192.4 and 188.6 J/g°C) and the control (221.1 J/g°C) samples. Clearly, the combined treatments performed in this study were greatly beneficial to the stability of shrimp muscle during frozen storage.

**Table 2:** Effect of different treatments on the changes in enthalpy change (Δ*H*, J/g°C) and glass transition temperature ($T_{\text{g}}\text{’}$, °C) of shrimp muscle determined by using a differential scanning calorimeter (DSC).

**Items**	**Control**	**STPP**	**STPP-VP**	**Trehalose**	**Trehalose-VP**
*ΔH* (J/g°C)	221.1 ± 1.1^c^	192.4 ± 1.3^b^	168.9 ± 1.0^a^	188.6 ± 1.6^b^	170.3 ± 1.3^a^
$T_{\text{g}}\text{’}$ (°C)	−69.1 ± 0.2^a^	−57.6 ± 0.2^b^	−53.4 ± 0.1^c^	−58.6 ± 0.3^b^	−54.4 ± 0.2^c^

The means with different lowercase letters in the same row were significantly different at P < 0.05.

Previously, several reports have described a close correlation between the stability of muscle products and their glass transition temperatures (27). Muscle products are comparatively stable when they are stored below or at the glass transition temperature (under a frozen amorphous glassy state), whereas the muscle is prone to chemical and physical deterioration when stored above the glass transition temperature for long periods. In the current study, the permeated STPP/trehalose might form crystalline hydrates and affect the rheological properties of water and hydrophilic components in muscle tissues. The concentrated water molecules by STPP/trehalose became kinetically immobilized to form a viscous state. Therefore, the water molecules could not participate in the deterioration reactions during frozen storage (8). The water molecules interacting with STPP/trehalose through hydrogen bonds reduced the energy variations (enthalpy changes, Δ*H* values) of muscle tissues during the freezing phase transition (28). Moreover, the proteins might be embedded in the glassy matrix formed by STPP/trehalose molecules during long-term storage. Therefore, the rearrangement, dissociation, and/or conformational changes, as well as the degradation reactions of proteins, were greatly inhibited or limited by the immobilization (27, 29, 30). Therefore, the combination treatments significantly increased the $T_{\text{g}}\text{’}$ values of shrimp muscle tissues, and the shrimp products could be stored at comparatively higher temperatures with longer shelf-life, greater stability, and limited physicochemical reactions during frozen storage.

## Conclusion

The present study aimed to evaluate the cryoprotective effects of vacuum permeation (VP) combined with tripolyphosphate (STPP) and trehalose soaking treatments on shrimp muscle proteins during frozen storage. The results indicated the thawing loss, springiness, chewiness, myofibrillar protein content, Ca^2+^-ATPase activity, and the histological microstructure of shrimp muscle pre-treated with STPP/trehalose-VP were maintained over 120 d of frozen storage, compared with the control and individual STPP/trehalose treatments. The vitrification mechanisms of shrimp muscle tissues stabilized by the combination treatments (enhanced $T_{\text{g}}\text{’}$ values) were further validated *via* thermodynamic properties analysis. The findings advanced the traditional application of phosphates and trehalose, providing an effective preservation method for frozen shrimp products.

## Data availability statement

The raw data supporting the conclusions of this article will be made available by the authors, without undue reservation.

## Author contributions

QD and CF: investigation, methodology, and formal analysis. HQ: conceptualization and writing—original draft. SB and SA: validation and writing—review editing. BZ: data curation, project administration, and writing—review editing. All authors contributed to the article and approved the submitted version.

## Funding

This study was funded by the Zhejiang Natural Science Foundation of China (No. LR21C200001), the Zhejiang Leading Training Program (2020R52027), the National Natural Science Foundation of China (No. 32072146), and the Fundamental Research Funds for Zhejiang Province (2021JD005).

## Conflict of interest

The authors declare that the research was conducted in the absence of any commercial or financial relationships that could be construed as a potential conflict of interest.

## Publisher's note

All claims expressed in this article are solely those of the authors and do not necessarily represent those of their affiliated organizations, or those of the publisher, the editors and the reviewers. Any product that may be evaluated in this article, or claim that may be made by its manufacturer, is not guaranteed or endorsed by the publisher.
