# Supplementary material for: Low-temperature vacuum permeation of sodium tripolyphosphate and trehalose suppresses the denaturation of myofibrillar proteins in peeled shrimp (Litopenaeus vannamei) during frozen storage
Source: Front Nutr. 2022 Oct 6;9:1012864. doi: 10.3389/fnut.2022.1012864 (PMC9583252; doi:10.3389/fnut.2022.1012864)
Supplement: Supplementary file 1 [file Table_1.pdf]

Supplementary Table S1 Effect of different vacuum degree (MPa) on the thawing loss of shrimp muscle tissues after 120 days of frozen storage.

Groups	Thawing loss (%) of shrimp treated with different vacuum conditions				
	0.03 MPa	0.05 MPa	0.07 MPa	0.09 MPa	0.11 MPa
STPP-VP	7.48 ± 0.16^{aC}	7.53 ± 0.14^{aC}	7.02 ± 0.17^{aB}	6.69 ± 0.10^{aA}	6.67 ± 0.15^{aA}
Trehalose-VP	7.79 ± 0.14^{aC}	7.81 ± 0.18^{aC}	7.18 ± 0.12^{aB}	6.70 ± 0.12^{aA}	6.59 ± 0.19^{aA}

The means with different lowercase letters in the same column were significantly different at $P < 0.05$, while the means with different uppercase letters in the same row were significantly different at $P < 0.05$.
